# Sporotrichosis-Associated Hospitalizations, United States, 2000–2013

**DOI:** 10.3201/eid2210.160671

**Published:** 2016-10

**Authors:** Jeremy A.W. Gold, Gordana Derado, Rajal K. Mody, Kaitlin Benedict

**Affiliations:** Centers for Disease Control and Prevention, Atlanta, Georgia, USA

**Keywords:** sporotrichosis, epidemiology, mycoses, fungi, hospitalization, United States, USA

## Abstract

To determine frequency and risk for sporotrichosis-associated hospitalizations, we analyzed the US 2000–2013 National (Nationwide) Inpatient Sample. An estimated 1,471 hospitalizations occurred (average annual rate 0.35/1 million persons). Hospitalizations were associated with HIV/AIDS, immune-mediated inflammatory diseases, and chronic obstructive pulmonary disease. Although rare, severe sporotrichosis should be considered for at-risk patients.

Sporotrichosis is a fungal disease caused by the *Sporothrix schenckii* species complex (*1*)*.* In the environment, *S. schenckii* is commonly associated with decaying plant matter, soil, and sphagnum moss (*2*). Infection usually occurs through cutaneous inoculation of the organism and typically is a disease of the skin, subcutaneous tissue, and lymph nodes. Less commonly, disseminated forms of disease can occur if infection spreads from primary to secondary body sites, and pulmonary disease can occur if conidia are inhaled (*3*). Extracutaneous sporotrichosis typically develops in persons with immunosuppression, chronic obstructive pulmonary disease (COPD), diabetes mellitus, or alcoholism (*2*). Infections are usually sporadic, but outbreaks have been associated with traumatic skin injury sustained during outdoor work or with zoonotic spread from infected animals (*4**,**5*). Considered rare in the United States, sporotrichosis is not a reportable disease, and most information about its epidemiology comes from outbreak investigations.

To develop nationally representative estimates and to assess underlying conditions associated with sporotrichosis-associated hospitalizations, we analyzed data from the Healthcare Cost and Utilization Project (HCUP). This family of databases, sponsored by the Agency for Healthcare Research and Quality, comprises the largest collection of publicly available all-payer healthcare data in the United States.

## The Study

The HCUP National (referred to as Nationwide before 2012) Inpatient Sample (NIS) is a database of hospital inpatient stays derived from billing data from ≈1,000 community hospitals (*6*). Data from the 2012 and 2013 NIS represent a 20% stratified sample of discharges from all participating hospitals; before 2012, the NIS contained all discharges from a sample of hospitals. For national estimates, discharges are assigned specific sampling weights based on hospital census region, rural/urban location, teaching status, bed size, and ownership. Weighted, the NIS estimates that >36 million discharges occur yearly.

We identified sporotrichosis-associated hospitalizations in the 2000–2013 NIS by using diagnosis code 117.1 from the International Classification of Diseases, Ninth Revision, Clinical Modification (ICD-9-CM). We created dichotomous variables to identify patients discharged with selected concurrent conditions by using ICD-9-CM codes for the following: HIV/AIDS (042); solid organ transplant or hematopoietic stem cell transplant (V42 [excluding V42.3–V42.5], 996.8); immune-mediated inflammatory diseases commonly treated with immunosuppressive medications including glucocorticoids and biological agents such as tumor necrosis factor-α (TNF-α) inhibitors: rheumatoid arthritis (714.0, 714.2), inflammatory bowel disease (555.xx, 556.xx), and psoriasis (696.0, 696.1, 696.8); diabetes mellitus (249.xx, 250.xx); alcohol use disorders and associated conditions (291.xx, 303.xx, 305.0, 535.3, 571.2, 571.3); and COPD (490, 491, 492, 494, 496). We categorized sporotrichosis-associated hospitalizations as lymphocutaneous, pulmonary, or arthritic sporotrichosis by using ICD-9-CM codes suggestive of lymphocutaneous disease (289.1, 289.3, 457.2, 681.00, 681.10, 682.0–682.9, 683, 686.9, 709.9), pulmonary disease (484.7, 486, 491.21, 493.22, 518.89, 786.2, 786.39), and infectious arthropathy (711.xx), respectively.

We obtained national estimates of sporotrichosis-associated hospitalizations and 95% CIs by applying the HCUP-supplied discharge weights (*7*) and examined them by age, sex, hospital region, season, presence of certain underlying conditions, in-hospital deaths, length of stay, and hospital charges by using SAS (SAS Institute, Cary, NC, USA) survey procedures. We calculated average annual overall rates and age-, sex-, and region-specific rates per 1 million persons by using population statistics from the US Census Bureau. We compared sporotrichosis-associated and non–sporotrichosis-associated hospitalizations by using the Rao-Scott χ^2^ test for categorical variables (*8*) and the Student *t*-test for continuous variables.

During 2000–2013, an estimated 1,471 (95% CI 1,245–1,696) sporotrichosis-associated hospitalizations occurred; the average annual rate was 0.35/1 million persons (95% CI 0.30–0.40) (Table). Yearly rates ranged from 0.19–0.47/1 million persons and showed no apparent trend (Figure). Sporotrichosis was listed as the primary patient diagnosis for 29.5% of all sporotrichosis-associated hospitalizations.

**Table:** Characteristics of sporotrichosis-associated hospitalizations versus non–sporotrichosis-associated hospitalizations, United States, 2000–2013*

Characteristic	Sporotrichosis-associated hospitalizations			Non–sporotrichosis-associated hospitalizations, %	p value
	No. hospitalizations (95% CI)	Average annual rate (95% CI)†	% Hospitalizations		
Overall no.	1,471 (1,245–1,696)	0.35 (0.30–0.40)		518,905,960	
Patient age group, y					<0.001
<18‡	–	–	–	16.6	
18–44	329 (240–419)	0.21 (0.15–0.26)	20.9	25.8	
45–64	599 (466–734)	0.57 (0.44–0.70)	40.8	23.0	
>65	451 (427–655)	1.01 (0.79–1.22)	36.8	34.6	
Mean patient age, y (SE)			58.1 (1.0)	47.9 (0.1)	<0.001
Patient sex					<0.001
M	831 (679–982)	0.40 (0.33–0.48)	56.4	41.4	
F	640 (513–767)	0.30 (0.24–0.36)	43.5	58.6	
Hospital region					<0.001
Northeast	118 (65–171)	0.15 (0.08–0.22)	8.0	19.4	
Midwest	328 (239–418)	0.35 (0.26–0.45)	22.3	22.9	
South	589 (471–706)	0.38 (0.31–0.46)	40.0	38.3	
West	436 (310–561)	0.45 (0.32–0.58)	29.6	19.4	
Season					0.006
Winter	257 (184–330)		17.5	23.2	
Spring	378 (289–467)		25.7	23.5	
Summer	371 (281–461)		25.2	23.2	
Autumn	349 (252–446)		23.7	22.8	
Unknown	116 (68–163)		7.9	7.3	
Underlying conditions					
HIV/AIDS	19 (0–42)		1.3	0.4	0.009
Transplant	19 (0–42)		1.3	0.7	0.289
Immune-mediated inflammatory disease	118 (66–170)		8.0	1.9	<0.001
Diabetes mellitus	285 (204–366)		19.4	17.7	0.294
Alcoholism	49 (16–83)		3.4	2.5	0.262
COPD	242 (149–335)		16.5	10.9	0.006
None of the above	877 (726–1,028)		59.6	69.3	
Patient deceased at discharge	49 (18–79)		3.3	2.0	<0.001
Mean length of hospital stay, d (SE)			6.9 (0.5)	4.6 (0)	<0.001
Mean charges, US$ (SE)			36,131 (4,290)	25,906 (166)	0.017
Disease form§					
Lymphocutaneous	641 (517–766)		43.6		
Pulmonary	160 (98–222)		10.9		
Arthritis	47 (15–80)		3.2		
Unclassified	623		45.5		

*Values are % or no. (%) unless otherwise indicated. COPD, chronic obstructive pulmonary disease. †Per 1 million persons. ‡Data for this age group not presented because of small sample size. §Categorized using codes from the International Classification of Diseases, Ninth Revision, Clinical Modification, suggestive of lymphocutaneous disease (289.1, 289.3, 457.2, 681.00, 681.10, 682.0–682.9, 683, 686.9, 709.9); pulmonary disease (484.7, 486, 491.21, 493.22, 518.89, 786.2, 786.39); and infectious arthropathy (711.xx).

**Figure:**
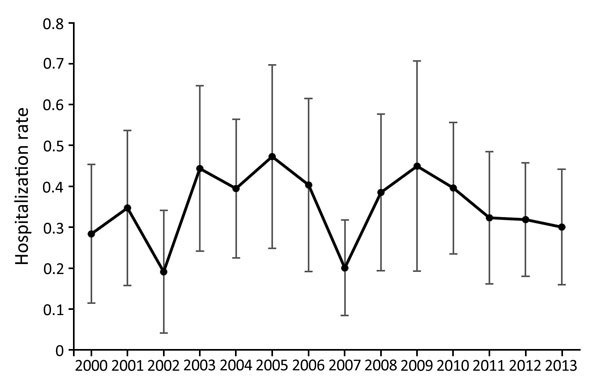
Annual rates of sporotrichosis-associated hospitalizations (no. hospitalizations/1 million persons), United States, 2000–2013. Error bars represent 95% CIs**.**

Sporotrichosis-associated hospitalizations occurred almost exclusively among adults, and rates were higher among men (average annual rate 0.40/1 million persons) than women (0.30/1 million). Rates were highest in the western (0.45/1 million) and lowest in the northeastern (0.15/1 million) United States. Nearly half (43.6%) of sporotrichosis-associated hospitalizations were for lymphocutaneous disease, but the clinical category of sporotrichal illness could not be identified for 42.3%. The discharge record diagnosis for sporotrichosis-associated hospitalizations was more likely than that for non–sporotrichosis-associated hospitalizations to mention HIV/AIDS (1.3% vs. 0.4%; p = 0.009), immune-mediated inflammatory disease (8.0% vs. 1.9%; p<0.001), or COPD (16.5% vs. 10.9%; p = 0.006), and the hospital stay was more likely to be longer (6.9 vs. 4.6 days; p<0.001) and costlier (mean charges $36,131 vs. $25,906; p = 0.017).

## Conclusions

Our update of the epidemiology of sporotrichosis-associated hospitalizations in the United States demonstrates that the annual incidence remains low, at an estimated 0.35 hospitalizations/1 million persons. To our knowledge, national data on sporotrichosis-associated hospitalizations have not been described since Reingold et al. reported an annual incidence of 2.4/1 million persons during 1980–1982 (*9*). Their estimate was based on a review of hospital discharge data from the Professional Activity Study of the Commission on Professional and Hospital Activities. Our data suggest that the current incidence of sporotrichosis-associated hospitalizations is lower than it was in the 1980s, which may be the result of increased awareness of sporotrichosis prevention methods (such as wearing long-sleeved clothing and protective gloves and avoiding sphagnum moss) among workers in higher risk occupations after several high-profile outbreaks of lymphocutaneous sporotrichosis in the 1980s (*10*). Our estimate is consistent with that obtained from population-based laboratory surveillance during 1992–1993 in the San Francisco Bay area (California, USA), which estimated a yearly sporotrichosis incidence of <1 case/1 million persons (*11*).

We found that slightly more sporotrichosis-associated hospitalizations occurred among men than women and among persons 18–64 years of age. These findings may reflect participation in outdoor activities that could expose persons to *Sporothrix *(*4*). Although most sporotrichosis outbreaks have occurred in midwestern and southern states, sporotrichosis-associated hospitalizations were most common in western states.

Using administrative data has limitations: ICD-9-CM codes may not capture all cases because of possible misclassification and do not distinguish the more common lymphocutaneous form from other, more severe, forms of sporotrichosis. This limitation may be alleviated in future analyses because codes in the International Classification of Diseases, Tenth Revision, do distinguish the different forms of sporotrichosis. Using ICD-9-CM codes indicative of unspecified lymphocutaneous disease, we classified nearly half of all sporotrichosis-associated hospitalizations as the lymphocutaneous form. However, many more cases of lymphocutaneous sporotrichosis not requiring patient hospitalization probably occur; during the largest recorded outbreak of lymphocutaneous sporotrichosis in the United States, only 20% of case-patients were hospitalized (*10*)*.*

Similar to previous reports describing factors that predispose persons to severe sporotrichosis, we found that HIV/AIDS and COPD were more commonly listed on discharge records for sporotrichosis-associated hospitalizations than for non–sporotrichosis-associated hospitalizations. However, factors associated with specific forms of sporotrichosis could not be assessed because of the small sample sizes for these subgroups. Our analysis showed that immune-mediated inflammatory diseases commonly treated with glucocorticoids and TNF-α inhibitors were 4 times more common among hospitalizations for sporotrichosis than hospitalizations for other reasons. The association between invasive mycoses and hypercortisolism has been well documented (*12*). No meaningful increase in sporotrichosis-associated hospitalizations during 2000–2013 was noted; however, as biological agents such as TNF-α inhibitors are being more frequently used to treat immune-mediated inflammatory diseases, and given their association with disseminated mycoses (*13**,**14*), there remains the potential that sporotrichosis-associated hospitalizations will increase in the future. Although severe forms of sporotrichosis are relatively rare, physicians should continue to be aware of potential for this disease in at-risk patients, particularly those who are receiving immunosuppressive agents and those with HIV/AIDS or COPD.

## References

[1] Marimon R, Cano J, Gené J, Sutton DA, Kawasaki M, Guarro J. *Sporothrix brasiliensis, S. globosa*, and *S. mexicana*, three new *Sporothrix* species of clinical interest. J Clin Microbiol. 2007;45:3198–206. 10.1128/JCM.00808-07 17687013 PMC2045377

[2] Barros MB, de Almeida Paes R, Schubach AO. *Sporothrix schenckii* and Sporotrichosis. Clin Microbiol Rev. 2011;24:633–54. 10.1128/CMR.00007-11 21976602 PMC3194828

[3] Kauffman CA, Bustamante B, Chapman SW, Pappas PG; Infectious Diseases Society of America. Clinical practice guidelines for the management of sporotrichosis: 2007 update by the Infectious Diseases Society of America. Clin Infect Dis. 2007;45:1255–65. 10.1086/522765 17968818

[4] Chakrabarti A, Bonifaz A, Gutierrez-Galhardo MC, Mochizuki T, Li S. Global epidemiology of sporotrichosis. Med Mycol. 2015;53:3–14. 10.1093/mmy/myu062 25526781

[5] Schubach AO, Schubach TM, Barros MB. Epidemic cat-transmitted sporotrichosis. N Engl J Med. 2005;353:1185–6. 10.1056/NEJMc051680 16162897

[6] Healthcare Cost and Utilization Project. National (Nationwide) Inpatient Sample. Rockville (MD): Agency for Healthcare Research and Quality; 2015 [cited 2015 Dec 1]. https://www.hcup-us.ahrq.gov/nisoverview.jsp

[7] Healthcare Cost and Utilization Project. Trend weights for 1993–2011 HCUP NIS data. Rockville (MD): Agency for Healthcare Research and Quality; 2011 [cited 2015 Dec 1]. http://www.hcup-us.ahrq.gov/db/nation/nis/trendwghts.jsp

[8] Rao JNK, Scott AJ. The analysis of categorical data from complex sample surveys: chi-squared tests for goodness of fit and independence in two-way tables. J Am Stat Assoc. 1981;76:221–30 . 10.1080/01621459.1981.10477633

[9] Reingold AL, Lu XD, Plikaytis BD, Ajello L. Systemic mycoses in the United States, 1980-1982. J Med Vet Mycol. 1986;24:433–6. 10.1080/02681218680000691 3572678

[10] Coles FB, Schuchat A, Hibbs JR, Kondracki SF, Salkin IF, Dixon DM, A multistate outbreak of sporotrichosis associated with sphagnum moss. Am J Epidemiol. 1992;136:475–87. 1415167 10.1093/oxfordjournals.aje.a116521

[11] Rees JR, Pinner RW, Hajjeh RA, Brandt ME, Reingold AL. The epidemiological features of invasive mycotic infections in the San Francisco Bay area, 1992-1993: results of population-based laboratory active surveillance. Clin Infect Dis. 1998;27:1138–47. 10.1093/clinids/27.5.1138 9827260

[12] Lionakis MS, Kontoyiannis DP. Glucocorticoids and invasive fungal infections. Lancet. 2003;362:1828–38. 10.1016/S0140-6736(03)14904-5 14654323

[13] Novosad SA, Winthrop KL. Beyond tumor necrosis factor inhibition: the expanding pipeline of biologic therapies for inflammatory diseases and their associated infectious sequelae. Clin Infect Dis. 2014;58:1587–98. 10.1093/cid/ciu104 24585557

[14] Ursini F, Russo E, Leporini C, Calabria M, Bruno C, Tripolino C, Lymphocutaneous sporotrichosis during treatment with anti-TNF-alpha monotherapy. Case Reports in Rheumatology. 2015;2015: 1–3. 10.1155/2015/614504 PMC4338406 25755904

